# Bruton's tyrosine kinase (Btk) inhibitor ibrutinib suppresses stem-like traits in ovarian cancer

**DOI:** 10.18632/oncotarget.3658

**Published:** 2015-03-26

**Authors:** Muhammad Ary Zucha, Alexander T.H. Wu, Wei-Hwa Lee, Liang-Shun Wang, Wan-Wan Lin, Chiou-Chung Yuan, Chi-Tai Yeh

**Affiliations:** ^1^ Graduate Institute of Medical Sciences, Taipei Medical University, Taipei, Taiwan; ^2^ Department of Obstetrics and Gynecology, Gadjah Mada University-Sardjito Central Hospital, Yogyakarta, Indonesia; ^3^ Graduate Institute of Medical Science and Technology, Taipei Medical University, Taipei, Taiwan; ^4^ Translational Research Laboratory, Cancer Center, Taipei Medical University Hospital, Taipei, Taiwan; ^5^ Department of Pathology, Taipei Medical University-Shuang Ho Hospital, Taipei, Taiwan; ^6^ Graduate Institute of Clinical Medicine, College of Medicine, Taipei Medical University, Taipei, Taiwan; ^7^ Obstetrics and Gynecology Department, Shuang-Ho Hospital, Taipei, Taiwan; ^8^ Department of Medical Research and Education, Taipei Medical University-Shuang Ho Hospital, Taipei, Taiwan

**Keywords:** ovarian cancer, cancer stem cell (CSC), spheroids, ibrutinib, Bruton's tyrosine kinase (Btk), cisplatin

## Abstract

According to a Prognoscan database, upregulation of Bruton's tyrosine kinase (Btk) is associated with low overall survival in ovarian cancer patients. We found that spheroids-forming ovarian cancer cell, which highly expressed cancer stem-like cell (CSC) markers and Btk, were cisplatin resistant. We next treated CSCs and non-CSCs by a combination of ibrutinib and cisplatin. We found that chemoresistance was dependent on Btk and JAK2/STAT3, which maintained CSC by inducing Sox-2 and prosurvival genes. We suggest that addition of ibrutinib to cisplatin may improve treatment outcome in ovarian cancer.

## INTRODUCTION

Ovarian cancer is the fourth most common malignancy in women and is the leading cause of death from gynecological cancers [1]. With an extremely high mortality rate, ovarian cancer is a public health problem worldwide. Ovarian cancer has typical clinical behavior. Even though 80% of ovarian patients have symptoms in early stage when the cancer is only limited in the ovary, the non-distinctive symptoms make more than 75% ovarian cancer patients diagnosed in late stage [1-3]. Ninety-two percent of ovarian cancer patients in stage I have a 5-year survival. However, patients diagnosed in the late stage have poor prognosis, with only 19% of 5-year survival rate for stage IV patients [1, 4]. Because ovarian cancer exhibits this clinical behavior, developing an effective treatment strategy for late-stage ovarian cancer patients is crucial. Although the current standard first-line treatment for late-stage ovarian cancer, which includes a combination of cytoreductive surgery and platinum-based chemotherapy, usually yields a multiyear survival rate, prolonged use of platinum-based chemotherapy usually induces drug resistance. Although a deeper understanding of this disease has been attained, relapse still occurs in 70% of patients 18 months after the first-line treatment [4, 5]. Therefore, it is crucial to develop a novel drug that effectively impacts on ovarian cancer especially that is resistant to current chemotherapy [4, 6].

Recent evidence has suggested that a small subset of ovarian cancer cells with cancer stem cell (CSC) properties can survive chemotherapy and play a major role in tumor relapse. Platinum-based chemotherapy effectively kills common cancer cells, but it fails to kill CSCs. CD44+/CD117+ cells isolated from human epithelial ovarian cancer show stem cell properties with high tumorigenicity *in vivo*, strong survival, and high resistance to several chemotherapeutic drugs, including 5-fluorouracil, carboplatin, and cisplatin. For CD117+ cells, the aforementioned properties are suggested to be due to the overexpression of ABCG2 and ABCB1 transporters, which can effectively efflux chemotherapeutic drugs [7]. Furthermore, because CSCs have self-renewal ability, they can promote tumor progression and clinical recurrence [5, 8, 9]. Therefore, pharmacologic targeting of CSCs is very promising [10].

Targeting signaling pathways that are specifically crucial in CSCs might be a useful strategy for developing a breakthrough treatment. Bruton's tyrosine kinase (Btk) is a nonreceptor tyrosine kinase that exhibits various modulatory effects in response to external stimuli. The Btk family can mediate downstream signaling pathways of G-protein coupled receptors, antigen receptors, and integrins to regulate cell growth, differentiation, and apoptosis [11]. B lymphocytes [12] and myeloid cells [13] with low Btk activity tend to undergo apoptosis and exhibit decreased proliferation, suggesting the importance of Btk in cell survival and growth pathways. Moreover, Btk inhibits Fas-ligand-mediated apoptosis, but induces Akt and NF-κB activation [14, 15]. In addition, another major pathway downstream of Btk that increases cancer progression is mediated by activation of signal transducer and activator of transcription 3 (STAT3). Although STAT3 has been proven to regulate CSCs in some cancers [16, 17], its involvement through Btk signaling in ovarian cancer remains unproven. Therefore, in this study, we explored the role of Btk in regulating ovarian CSCs; particularly, we examined the link between Btk regulation and the STAT3 pathway. Furthermore, according to the PrognoScan bioinformatics database (http://www.abren.net/PrognoScan/), the overall survival rate of patients with Btk overexpression is significantly lower than that of patients with low Btk expression. We here demonstrated that ovarian cancer spheroids that represent CSCs are highly resistant to cisplatin, and this resistance is attributable to the overexpression of Btk signaling. Btk silencing effectively reduced the expression of the Janus kinase 2 (JAK2)/STAT3 pathway, which in turn suppressed the survival of cancer cells through Sox-2 and BCL-XL genes and, finally, restored responsiveness to cisplatin. In addition, we proved that ibrutinib as an adjunct to cisplatin has synergistic effects on cancer cells, indicating that this drug can improve the clinical response to cisplatin in the future.

## RESULTS

### Btk is a histological biomarker and a prognostic predictor of ovarian cancer

**Figure 1:**
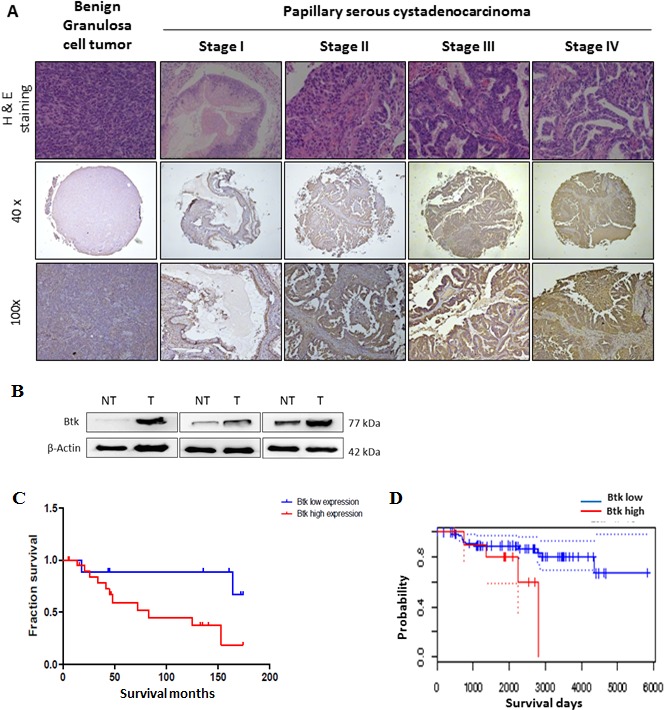
High expression of Btk in human ovarian tumors is associated with a poor cancer prognosis (**A**)Immunohistochemistry staining was performed to identify the expression of Btk in ovarian cancer tissue microarrays. Representative images of immunostained tissues from different stages of ovarian cancer patients and benign ovarian patients are presented. (**B**) Three ovarian tissue specimens were randomly selected and subjected to microdissection to separate the tumor and nontumor parts. The expression of Btk was assessed using western blotting. Our data showed that the expression of Btk in tumor samples was consistently higher in the tumor parts than in surrounding nontumor parts. (**C**) Immunohistochemically stained malignant samples were categorized into two groups and compared with the overall survival rate. (**D**) The Kaplan–Meier graph was consistent with survival data from the PrognoScan database, revealing that the expression of Btk is correlated with lower overall survival.

Tissue samples from ovarian cancer patients (n = 50) were studied to determine the expression of Btk in correlation with clinical parameters. The results of immunohistochemical (IHC) staining of Btk and histotypes of all recruited ovarian cancer patients are summarized in Table 1. We categorized the IHC staining results into the following three groups as described previously [18]: no staining (n = 5), weak or focal staining (n = 21), and moderate or intense staining (n = 24). IHC staining of tissue arrays showed that the expression of Btk was high in malignant cells and in line with disease progression (Fig. 1A, Table 1). Compared with the tissues from patients in the early stage, those from patients in the late stage exhibited more intense Btk staining. Granulosa cells that originated from the sex cord showed negative staining for Btk, whereas malignant cells consistently expressed Btk (Fig. 1A). We also found that Btk expression was related to metastasis (χ^2^=4.146; p=0.042) and clinical stage (χ^2^=4.080; p=0.043) (Table 2). Immunoblotting data showed that the expression of Btk was consistently higher in tumor parts than in nontumor parts (Fig. 1B). In addition, we observed that patients with moderate or intense staining of Btk had a significantly lower survival rate than those with no or weak or focal staining of Btk (Fig. 1C). Our Kaplan–Meier graph was consistent with survival data from the PrognoScan database (Fig. 1D). According to the bioinformatics database provided by PrognoScan, Btk overexpression was correlated with a lower overall survival rate in ovarian cancer. All of these results suggested that Btk is specifically expressed in malignant cells and its expression is in line with disease progression. We used Cox proportional hazard model to determine the relationship of four parameters (age, metastasis, stage and Btk expression) to overall survival in malignant ovarian tumor patients with surgical resection. Among these factors, only high Btk expression was related to unfavorable overall survival (hazard risk=1.115; 95% confidence interval=1.047 – 2.293) (Table 3). Therefore, Btk might be a potential histological biomarker and a prognostic predictor of overall survival in ovarian cancer patients.

**Table 1:** Immunohistochemical detection of Btk in ovarian tissue array (n=50)

Samples	No. of cases	Immunohistochemical staining		
		No staining	Weak/Focal staining^*^	Moderate/Intense staining^#^
Age (mean ± SD)		47.00 ± 8.15	44.17 ± 20.55	52.39 ± 12.19
Stage: *n* (%)				
Benign tumor	11	2 (18.18)	6 (54.54)	3 (27.27)
Borderline tumor	6	2 (33.33)	3 (50)	1 (16.67)
Malignant tumor				
I	6		6 (100)	
II	15	1 (6.67)	4 (26.67)	10 (66.67)
III	8		2 (25)	6 (75)
IV	4			4 (100)
Histotypes: *n* (%)				
Epithelial tumors				
Mucinous borderline	1		1 (100)	
Serous				
Benign	1		1 (100)	
Borderline	2	1 (50)	1 (50)	
Malignant	15		2 (13.33)	13 (86.67)
Undifferentiated	1		1 (100)	
Mullerian	2		2 (100)	
Endometrioid	5		1 (20)	4 (80)
Clear cell carcinoma	3		2 (66.67)	1 (33.33)
Sex cord stromal tumor	5	1 (20)	3 (60)	1 (20)
Germ cell tumor	5	1 (20)	3 (60)	1 (20)
Stromal tumors	6	2 (33.33)	3 (50)	1 (16.67)
Metastasis to ovary	4		1 (25)	3 (75)

* Diffuse weak or focal intense staining

# Moderate or intense staining of >80% of tissue

**Table 2:** Correlation between clinicopathologic variables and immunohistochemical expression of Btk in malignant ovarian cancer patients

Clinical parameters	Variables	No of patients	Btk expression		χ2	Odds ratio	*p* value
			Lown n (%)	Highn n (%)			
Age (years)	≥60	8	4 (50)	4 (50)	0.498	1.778	0.4806
	<60	25	9 (36)	16 (64)			
Metastasis	No	24	12 (50)	12 (50)	4.146	8.00	0.0417
	Yes	9	1 (11.11)	8 (88.89)			
Stage	I/II	21	11 (52.38)	10 (47.62)	4.080	5.50	0.0434
	III/IV	12	2 (16.67)	10 (83.33)			

**Table 3:** Cox multivariate proportional hazard model of independent predictors on overall survival

Variable	Hazard ratio	95% confidence interval	*P* value
Btk expression (high vs low)	1.115	1.047 – 2.293	0.032

### Ovarian CSCs contribute to cisplatin resistance

**Figure 2:**
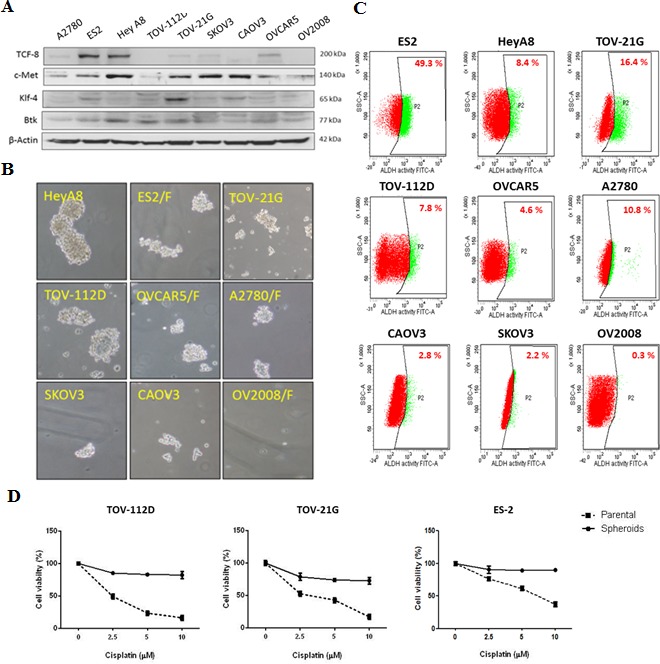
Ovarian spheroids are highly resistant to cisplatin (**A**) The protein levels of endogenous genes for stem cell and EMT regulation in nine ovarian cancer cell lines were surveyed. (B, C) The ALDH1 activity (**B**) and spheroid formation (**C**) of the cell lines were determined. (**D**) Cell viability after treatment with cisplatin was compared between spheroids and parental cells. Spheroids and parental cells were treated with increasing doses of cisplatin (2.5–10 μM) for 48 h, and the viability of these cells was assessed. Data are presented as the mean ± SD of triplicate experiments.

The proportion of CSCs in ovarian cancer was reported to be a prognostic predictor and can be detected according to the aldehyde dehydrogenase 1 (ALDH1) activity [20]. To understand the importance of ovarian CSCs in cisplatin resistance, we initially determined the molecular characteristics of stemness and epithelial-to-mesenchymal transition (EMT)-related genes, including Klf-4, c-Met, and TCF-8, in nine ovarian cancer cell lines. These cancer cell lines, according to their platinum resistance, have been categorized as highly resistant, moderately resistant, or responsive to platinum-based therapy. ES-2 [21], Hey-A8 [22, 23], TOV-21G [24], and TOV-112D [25] are highly resistant to chemotherapy, whereas CAOV-3, SKOV-3, OVCAR-5, and A2780 are moderately resistant to chemotherapy [23, 25, 26]. OV-2008, with high responsiveness to cisplatin, was the least malignant cell line in this study [26]. We observed that cells from the more malignant cell lines ES-2, TOV-21G, and Hey-A8 had high expression levels of Klf-4, c-Met, and TCF-8 (Fig. 2A). By contrast, OV-2008 cells exhibited low expression levels of Klf-4, c-Met, and TCF-8 (Fig. 2A). Next, we analyzed the phenotype of spheroid formation in these cancer cell lines. As expected, cells from more malignant cell lines such as ES-2 and Hey-A8 had greater ability to form ovarian spheroids than did OV-2008 cells (Fig. 2B). Because ALDH1 activity is a conventional marker for CSC [27], we examined the expression of ALDH1 as an index of the proportion of CSCs. Flow cytometry data revealed that ES-2, TOV-21G, and Hey-A8 cells that expressed high levels of TCF-8, Klf-4, and c-Met had higher proportion of ALDH1high cells (Fig. 2C), indicating the high proportion of CSCs. Finally, to study the importance of ovarian CSCs in chemoresistance, we generated spheroids from several parental cell lines and then compared the responsiveness to cisplatin between spheroids and parental cells. According to a cell viability assay conducted after treatment with cisplatin, compared with their respective parental cells, spheroids of the TOV-112D, TOV-21G, and ES-2 cell lines were more resistant to cisplatin (Fig. 2D). Treatment with cisplatin at concentrations up to 10 μM resulted in cell death rates of only 10%–20% in spheroids of the TOV-112D, TOV-21G, and ES-2 cell lines, and the IC50 values of cisplatin for these spheroids were 29.28, 17.82, and 57.16 μM, respectively. By contrast, the IC50 values of cisplatin for parental cells of the TOV-112D, TOV-21G, and ES-2 lines were 4.00, 4.82, and 9.84 μM, respectively. These high IC50 values indicated that, compared with the parental cells, spheroids exhibited high resistance to cisplatin. Thus, these findings suggest that CSCs play a major role in cisplatin resistance.

### Ovarian CSCs express Btk signaling pathway in high levels

**Figure 3:**
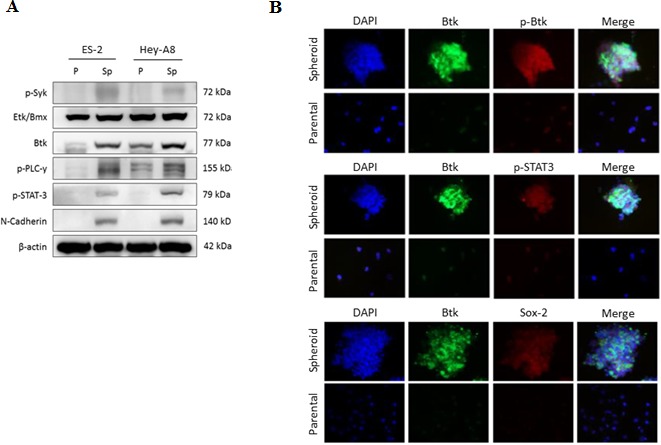
Ovarian spheroids enriched with CSCs highly express Btk signaling (**A**) The protein levels of Btk, Etk, Syk, p-PLCγ2, p-STAT3, and N-cadherin in parental (p) and spheroids (Sp) of ES-2 and Hey-A8 cells were assessed using western blotting. (**B**) *In situ* expression of Btk signaling was elucidated using 3D immunofluorescent staining. Antibodies against Btk, p-Btk, p-STAT3, and Sox-2 were used. Btk signaling was highly expressed and activated in ovarian spheroids, indicating the importance of Btk signaling in ovarian cancer stemness regulation.

Btk is nonreceptor tyrosine kinase that can be modulated by several upstream signaling pathways, including the spleen tyrosine kinase (Syk) pathway. Deregulation of the Syk–Btk axis has been implicated in certain hematological malignancies and immunological diseases [28]. Syk enhances Btk activity on several downstream proteins, such as phospholipase C gamma 2 (PLCγ2) [29] and STAT3 [11]. We observed that ovarian cancer spheroids originating from ES-2 cells, which overexpressed Sox-2 and N-cadherin, had high Btk pathway activation. In addition, Btk signaling in such spheroids might also follow the Syk–Btk–PLCγ2 axis, because our immunoblotting data revealed that spheroids of ES-2 and Hey-A8 cells expressed higher levels of Syk, Btk, and PLCγ2 than did the respective parental cells (Fig. 3A). In addition to PLCγ2 activation, STAT3 was upregulated in these spheroids (Fig. 3A). Etk is another member of the BTK family [11]. The significantly higher expression of Btk than of Etk indicated that the expression of CSCs is highly regulated by Btk. Therefore, the upregulation of N-cadherin in ovarian CSCs [30] was consistent with the expression of Btk. Moreover, microscopic immunofluorescence analysis confirmed the differences in the Btk, p-Btk, p-STAT3, and Sox-2 expression profiles between spheroids and parental cells from the ES-2 cell line (Fig. 3B). Altogether, these results proved our hypothesis that ovarian CSCs play a major role in cisplatin resistance and that Btk signaling is crucial for regulating ovarian CSCs.

### Overexpression of Btk promotes ovarian cancer survival and cisplatin resistance

**Figure 4:**
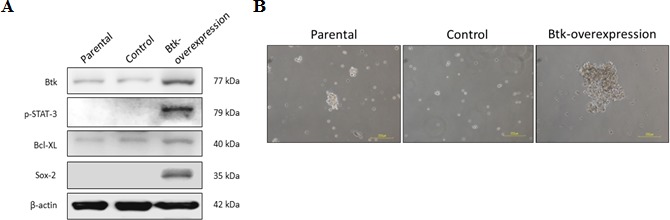
Overexpression of Btk promotes STAT3 pathway activation and causes cisplatin resistance For the gain-of-function study, a Btk-overexpression clone was generated from OV-2008, a benign cell line that exhibits low Btk expression. (**A**) The expression of Btk and its downstream proteins was compared with that of control non-Btk-overexpressing cells by using immunoblotting. The overexpression of Btk promoted STAT3 pathway activation. Consequently, downstream effectors, such as Sox-2 and Bcl-XL, were upregulated. (**B**) After Btk was overexpressed, cells could easily form spheroids that represented the enriched CSC population.

**Figure 5:**
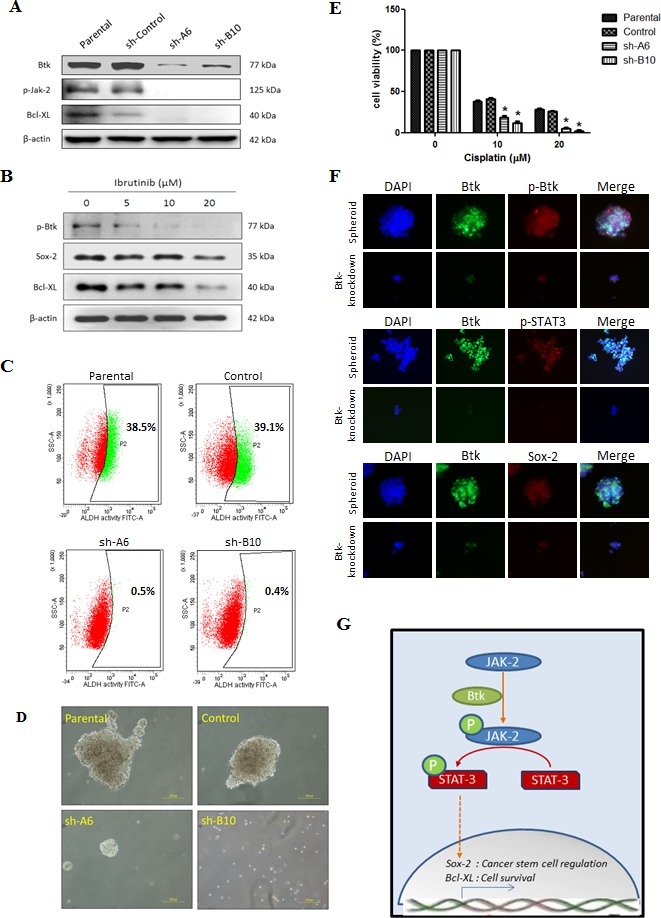
Btk inhibition targets CSCs and reduces their survival against cisplatin Gene silencing targeting Btk was achieved by transfecting cells with sh-RNA, namely sh-A6 and sh-B10. (**A**) ES-2 cells that endogenously overexpress Btk were knocked down. Btk knockdown leads to the downregulation of the JAK-2 pathway. (**B**) Ibrutinib (5–20 μM) was administered to ES-2 cells for 48 h, and its effect on STAT-3 target genes was then determined. (**C**) ALDH1 activity was compared between parental and Btk-silenced ES-2 cells. (**D**) After Btk knockdown, the ability to form spheroids was markedly lower in Btk-silenced ES-2 cells than in parental cells. (**E**) The chemosensitivity against cisplatin after Btk silencing was compared using an SRB assay. (**F**) *In situ* expression of Btk, STAT-3, and Sox-2 were elucidated using 3D immunofluorescent staining in Btk-knocked down ES-2 cells. (**G**) The proposed signal pathways of the Btk–STAT3 axis, which is involved in cancer stemness and cancer cell survival, are shown.

The importance of Btk in mediating the survival of ovarian cancer cells remains unclear. We proposed that Btk mediates chemoresistance through the regulation of ovarian CSCs. To prove our hypothesis, we performed gain-of-function (Fig. 4) and loss-of-function studies (Fig. 5). We overexpressed Btk in OV-2008 cells, which are primarily responsive to cisplatin, and observed that the overexpression of Btk promoted STAT3 activation. Consequently, several downstream effectors of STAT3, such as Sox-2 and Bcl-XL, were upregulated (Fig. 4A). Because of the high stemness gene regulation in OV-2008 cells overexpressing Btk, these cells easily formed spheroids and enriched the CSC population (Fig. 4B).

In loss-of-function studies, we found that Btk knockdown reduced the expression of JAK2 and STAT3 targets such as BCL-XL (Fig. 5A). The results of gene silencing experiments were comparable to those after treatment with ibrutinib (Fig. 5B). We observed that treatment with ibrutinib for 48 h concentration-dependently reduced Btk phosphorylation as well as Sox-2 and Bcl-XL protein expression. Subsequently, Btk silencing reduced the self-renewal ability of ES-2 cells and reduced the population of cells with high ALDH1 activity. This reduction in the population (Fig. 5C) and decrease in spheroid-forming ability (Fig. 5D) after Btk silencing indicated the suppression of ovarian CSCs. In addition, we proved that after Btk knockdown, ES-2 cells became more sensitive to cisplatin (Fig. 5E). Btk-silenced cells were compared with control cells following 48 h of treatment with cisplatin at concentrations ranging from 2.5 to 20 μM. A significant difference in cytotoxicity was observed in groups treated with cisplatin at 10 and 20 μM. Furthermore, we determined the expression of Btk in spheroids in situ. Human ovarian cancer spheroids were developed from parental and Btk-knocked-down ES-2 cells. We observed that the expression of Btk and phosphorylated Btk was markedly higher in the ES-2 spheroids than in the knocked-down cells. Accordingly, ES-2 spheroids exhibited high expression of p-STAT3 and Sox-2 (Fig. 5F). A summary of our proposed mechanism is presented in Fig. 5G.

### Cisplatin–ibrutinib combination has beneficial effects in eliminating ovarian cancer cells

**Figure 6:**
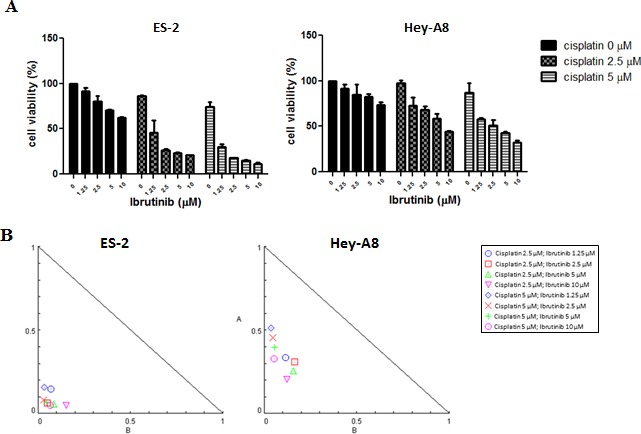
Btk inhibitor potentiates effects of cisplatin in eliminating ovarian cancer cells (**A**) Ibrutinib and cisplatin were coadministered in various concentrations in ES-2 and Hey-A8 cells, and 48 h later, viable cells were quantified using an SRB assay. (**B**) Combinational effects were studied using CompuSyn software that employs the Chou–Talalay algorithm for drug combination. Combinational effects are presented as the combination index (CI), where CI < 1 indicates synergism (inside triangle), CI = 1 indicates an additive effect, and CI > 1 (outside triangle) indicates antagonism. The cisplatin–ibrutinib combination had a beneficial effect on cytotoxicity because most CIs of both drugs were <1; thus, they demonstrated synergism.

A sulforhodamine-B (SRB) assay was performed to determine cell viability after treatment with cisplatin only, ibrutinib monotherapy, or a cisplatin–ibrutinib combination. In addition, isobologram or combination analysis was performed using CompuSyn software to study the combined effect of the two drugs. The cisplatin–ibrutinib combination had synergistic or additive effects on serous (Hey-A8) and clear cell types (ES-2) of ovarian cancers (Fig. 6A and 6B). The synergistic effects that were evident in most of the combination regimens indicated that the Btk inhibitor ibrutinib can sensitize cancer cells to platinum, whereas the additive effects indicated that both drugs independently eliminated cancer cells. Moreover, ibrutinib may be a potential drug candidate for overcoming platinum resistance in clear cell carcinoma and malignant cystadenocarcinoma, which are the most malignant and most prevalent subtypes, respectively.

## DISCUSSION

Ovarian cancer remains the most lethal gynecological malignancy worldwide. A challenge in the management of ovarian cancer is increasing treatment response to current standard chemotherapy. A large-scale randomized trial showed that in the advanced stage of the disease, cisplatin alone does not improve overall survival because of low responsiveness. Moreover, >55% of cancer patients in the trial relapsed after completing standard chemotherapy alone, and 72% of cancer patients relapsed after debulking surgery and platinum-based chemotherapy [31]. However, cisplatin, as the first-line chemotherapeutic agent for ovarian cancer, is the most widely used chemotherapy drug. Therefore, we investigated a strategy for potentiating the effect of cisplatin so that it can be easily translated in clinical settings. In addition, compared with other platinum-based chemotherapeutic drugs, cisplatin causes less myelosuppression, and, thus, combining it with other drugs is more feasible [32].

Although discovered decades ago, cisplatin is still widely used to treat several cancers, including ovarian cancer. Cisplatin functions by forming intrastrand cross-links with the target DNA, triggering necrosis or apoptosis. However, several mechanisms may help cancer cells evade cellular death induced by platinum [33]. Primarily, approximately 70%–90% of platinum resistance is caused by reduced platinum uptake or increased efflux [34]. CSCs overexpress ABC transporter, resulting in platinum efflux, and have a self-renewal ability, which triggers relapse. CSC population, which we measured on the basis of ALDH1 expression by using flow cytometry, may become a major tool for predicting prognosis. Correlations of ALDH1 activity with CSCs and poor clinical outcomes were previously reported in breast cancer [35]. In a gynecological malignancy, cisplatin resistance was correlated with CSC population, which was recognized according to the ALDH1 activity of CSCs [36]. Ovarian cancer patients with high ALDH1 activity have a significantly poor overall survival rate [20]. We surveyed the stemness regulator genotypes, spheroid formation ability, and ALDH1 activity of nine ovarian cancer cell lines and found that ES-2 and Hey-A8, as highly platinum-resistant cell lines, consistently exhibited high levels of ALDH1 activity. ES-2 was originated from clear cell carcinoma, a histological subtype of ovarian cancer with a poor prognosis [37], and this poor prognosis may be in line with the proportion of CSCs. In addition, we proved that ovarian CSCs are the major source of tumor development and resistance to chemotherapy because ovarian spheroids have higher resistance to cisplatin than do parental cells (Fig. 2). Therefore, the proposed application of ALDH1 activity as a prognostic predictor in ovarian cancer patients may be mediated by the chemoresistance properties of ovarian CSCs.

Spheroid formation is a method through which CSCs are enriched in various tumors. A spheroid is enriched with CSCs because it is formed through the self-renewal ability of CSCs [38, 39]. “Sphere-forming cells” or spheroids are commonly found within the ascitic fluid from late-stage ovarian cancer patients and become the source of metastasis because of high tumorigenicity [40]. In a previous study, 2000 cells from spheroids transplanted through xenografting triggered tumorigenesis, whereas 10^5^ wild-type cells were required to trigger tumorigenesis [41]. Pharmacological targeting of CSCs is very promising [10]. Inhibition of alkaline phosphatase by levamisole disrupted self-renewal ability of ovarian CSCs and suppressed tumor growth *in vivo* [42]. We confirmed that ovarian spheroids have higher resistance to cisplatin than do non-CSCs. However, we found that ovarian spheroids that were enriched with ovarian CSCs exhibited high Btk signaling (Fig. 3). Therefore, we determined the importance of Btk signaling in ovarian cancer and the possible application of the novel Btk inhibitor ibrutinib in ovarian cancer therapy. We examined the downstream effectors of the Btk pathway that play major roles in the maintenance and self-renewal of CSCs. After silencing Btk gene in ovarian cancer cell lines, we observed that the JAK2 pathway was downregulated (Fig. 5A). Therefore, expression levels of several effectors of STAT3, such as Bcl-XL and Sox-2, were diminished. Bcl-XL is a major cell cycle regulator (prosurvival), and its upregulation leads to increased cell growth [43]. Therefore, suppression of Bcl-XL can promote cell death. We showed that the expression of Sox-2 can be decreased through Btk silencing and can be upregulated through a Btk gain-of-function strategy. Furthermore, we confirmed that ibrutinib can concentration-dependently reduce the expression of Sox-2. Consequently, Btk silencing reduced the self-renewal ability of ovarian cancer spheroids (Fig. 5D). Moreover, administration of ibrutinib reversed chemosensitivity *in vitro*. Clear cell carcinoma cells, as one of the most malignant subtypes [37], regained chemosensitivity after Btk knockdown.

We observed the beneficial effect of ibrutinib in combination with cisplatin. Cisplatin was used in this study because it is the most widely used drug in conventional chemotherapy and it causes lower myelosuppression compared with other platinum-based chemotherapeutic drugs. Administering the Btk inhibitor ibrutinib exerted synergistic effects on cisplatin (Fig. 6). For combination analysis, we used the high-grade clear cell carcinoma ES-2 cell line, which is among the subtypes with the poorest prognosis, and the serous cystadenocarcinoma Hey-A8 cell line, which is the most prevalent subtype. We demonstrated that cisplatin–ibrutinib combination therapy had a significant effect on the elimination of cancer cells. Our data indicate the importance of using combination therapy to eradicate CSCs and non-CSCs. Clear cell carcinoma has been described as a prognostic factor for ovarian cancer. Patients with a clear cell carcinoma subtype easily develop chemoresistance and relapse. Thus, they have a poor prognosis [37]. According to our results, we believe that inhibition of the Btk pathway could be an effective strategy for overcoming platinum resistance. We also believe that the Btk inhibitor ibrutinib, of which the safety and efficacy in treating blood malignancies have been determined satisfactory in phase III clinical trials, can be applied in clinical settings [44]. However, the efficacy of ibrutinib in ovarian cancer therapy has never been studied. Collectively, our results indicate that administering ibrutinib, as a Btk inhibitor, may facilitate sensitizing ovarian cancer cells to cisplatin through inhibition of the JAK2 pathway.

## CONCLUSION

The present study is the first to report the importance of Btk in the chemoresistance and metastasis of ovarian cancer. The specific expression of Btk in ovarian malignancy may be useful as a novel histological biomarker. We showed that chemoresistant ovarian cancer cell lines highly expressed CSC regulatory genes. In addition, ovarian spheroids enriched with CSCs were more resistant to cisplatin when the Btk signaling pathway was activated. This result supports the concept of CSCs in chemoresistance and indicates that Btk inhibitors can be used as novel CSC-targeting drugs in ovarian cancer treatment. We demonstrated the beneficial effect of the Btk inhibitor ibrutinib in ovarian cancer treatment. Ibrutinib in combination with cisplatin had synergistic effects on chemotherapy. Btk plays crucial roles in regulating ovarian CSCs through JAK2/STAT3 activation. We proved that Btk inhibition through Btk gene silencing can affect CSC properties related to responsiveness to cisplatin. Altogether, our findings suggest that Btk is crucial in ovarian cancer chemoresistance. In addition, the Btk inhibitor ibrutinib may be beneficial as an adjunct for overcoming platinum resistance in ovarian cancer.

## MATERIALS AND METHODS

### Human tissue studies

Clinical samples were collected from Taipei Medical University-Joint Biobank (Taipei, Taiwan). All of the patients gave signed, informed consent for their tissues to be used for scientific research. Recommendations of the Declaration of Helsinki for biomedical research were also followed to get the approval by Joint Institutional Review Board (JIRB) of the Taipei Medical University (approval number: 201411003). Tissue array from 50 patients in different clinical stages were prepared for immunohistological analysis (Superbiochips, #CJ2). Antibody against Btk (1:400, SC-81159, Santa Cruz, USA) was used according to the standard immunohistochemistry protocol. A similar dilution of the control mouse IgG was applied as a negative control. The expression of Btk was then evaluated and confirmed by two pathologists. According to the expression of Btk, samples were categorized into the following groups: no staining, weak or focal staining, and moderate or intense staining. Samples with weak diffuse staining were considered as weak staining, whereas focal staining was defined as intense staining limited to a focal area. Staining was considered moderate or intense when the expression of Btk exceeded 80% [18]. Western blotting was performed to determine the Btk protein level in the tumor part relative to that in the nontumor part. The immunoblotting membrane was incubated with a primary antibody against Btk (1:600, SC-81159, Santa Cruz) and β-actin (1:5000, Abcam, UK).

### Culture of ovarian cancer cell line

The human ovarian cancer cell lines A2780, Hey A8, ES-2, SKOV-3, CAOV-3, TOV-112D, TOV-21G, OVCAR-5, and OV-2008 were provided by the American Type Culture Collection. All nine cell lines were maintained in the McCoy 5A medium (Gibco, Life Technologies, USA) with 10% fetal bovine serum in a standard condition. Spheroids were originated from parental cells in a stem cell medium according to an established protocol (Nutristem-XF, Biological Industries, Israel) [19]. A third generation of spheroids was collected to obtain highly pure enriched CSCs for further study.

### Assay for relative cell number

The cell lines were seeded in 96-well plates (3.5 × 10^5^ cells/well). After treatment, the cells were cultured for 48 h and the relative cell number was calculated using a SRB reagent according to the manufacturer's protocol (Sigma, USA). The viability of nonattached cells in ovarian spheroids was quantified using Alamar blue staining (Life Technologies, USA).

### Western blotting

Western blotting was performed using a standard method. The membrane was incubated with primary antibodies (list of antibodies is available in Supplementary material).

### Flow cytometry

The ALDH activity of the nine ovarian cancer cell lines was detected using the ALDEFLUOR assay kit (StemCell Technologies, USA) according to the standard protocol. Cells were suspended in the ALDEFLUOR assay buffer containing an ALDH substrate and incubated for 1 h at 37°C. Flow cytometry was performed using BD Fortessa (BD Biosciences, USA), and data were analyzed using BD software.

### Ovarian cancer spheroid formation

Cancer cell spheroids were generated according to a previously described protocol [19]. Parental cells (approximately 10^5^) were seeded on a nonattached vessel with a serum-free cell medium containing growth factors suitable for CSC enrichment. Non-stem cells died because of starvation, whereas CSCs were alive and aggregated to form spheroids. At least three passages were performed to obtain highly pure CSCs. The cells were seeded in a 25T flask and supplemented with a stem cell medium (Nutristem, Biological Industries, Israel) at 37°C in a 5% CO2 incubator for 2 days when the spheroids were clearly visible under a microscope.

### Btk gene-function analysis in ovarian cancer cell lines

The gain- and loss-of function of Btk in ovarian cancer cell lines were studied using commercially available systems. For gain-of-function study, the open-reading-frame of Btk (gene accession No. NM_000061) was cloned in pEZ-Lv105 expression vector (Cat. No. EX-A0534-Lv105, OmicsLinkTM Expression clone, GeneCoepia, USA). Btk gene-silencing shRNA mir sets (expression Arrest GIPZ lentiviral shRNA mir) were purchased from Thermo Scientific (USA). Two clones were found effectively silenced Btk expression, A6 (clone ID, V2LHS-89195) and B10 (V3LHS-639151) and non-silencing verified negative control (RHS4346) was used as control. The production of lentiviral particles (for both gain and loss of function studies) were carried out according to vendor's instructions and under strict adherence of practice guidelines in certified BSL-2 laboratory in The Integrated Laboratories for Translational Medicine, Taipei Medical University.

### Immunofluorescent staining analysis

Ovarian spheroids were fixed with paraformaldehyde and probed with primary antibodies against Btk, pBtk, pSTAT3, and Sox-2 (primary antibodies are listed in Supplementary material). A fluorophore-conjugated secondary antibody was used to observe the positive signal under confocal microcopy. DAPI was used to stain the nuclei of viable cells.

### Drug combination analysis

The cells were seeded onto 96 well-plates (3 × 103 cells/well)and subsequently treated with cisplatin for 48 h (abiplatin injection, Pharmachemie BV, the Netherlands) and a Btk inhibitor (ibrutinib, Cellagen Technology, USA), and viable cells were quantified using an SRB assay. To analyze the possible combined effects of cisplatin and ibrutinib, CompuSyn software was employed after the standard Chou–Talalay algorithm was used for combination analysis.

### Statistical analysis

Data are expressed as the mean ± standard deviation (SD). P values <0.05 were considered significant, and the level of confidence was set at 95%. The survival probability was measured with Kaplan-Meier method. Independent predictors associated with overall survival were analyzed with Stepwise Cox proportional hazard models. Statistical analyses were performed using GraphPad Prism (GraphPad Software Inc., USA) and PASW Statistics 18 (SPSS Ltd., Hong Kong).

## SUPPLEMENTARY MATERIAL AND TABLE


