# Supplementary material for: Bruton's tyrosine kinase (Btk) inhibitor ibrutinib suppresses stem-like traits in ovarian cancer
Source: Oncotarget. 2015 Mar 26;6(15):13255–68. doi: 10.18632/oncotarget.3658 (PMC4537012; doi:10.18632/oncotarget.3658)
Supplement: Supplementary file 1 [file oncotarget-06-13255-s001.pdf]

Bruton's tyrosine kinase (Btk) inhibitor ibrutinib suppresses stem-like traits in ovarian cancer

Supplementary Material

Supplemental table 1: List of antibody and application

Antibody	Catalogue number	Company	Diluton	Application
Btk	sc-28387	Santa Cruz	1:700 (WB)<br>1:400 (IF, IHC)	WB, IF, IHC
Syk	#2710	Cell Signaling	1:1000	WB
p-PLC-ó	#2821P	Cell Signaling	1:1000	WB
p-Jak-2	#4406P	Cell Signaling	1:1000	WB
Klf-4	#4038	Cell Signaling	1:1000	WB
c-Met	#8198	Cell Signaling	1:1000	WB
TCF-8	#3396	Cell Signaling	1:1000	WB
p-STAT-3	#9145P	Cell Signaling	1:1000 (WB)<br>1:400 (IF)	WB, IF
Sox-2	#3579	Cell Signaling	1:1000 (WB)<br>1:400 (IF)	WB, IF
Bcl-XL	E021061	Enogene	1:1000	WB
N-Cadherin	#4061	Genetex	1:1000	WB
â-actin	8226	Abcam	1:5000	WB

WB: Western blotting; IF : immunofluorescent staining; IHC: immunohistochemical staining
